# Comparative Evaluation of Periprostatic Nerve Block and Diclofenac Patch in Transrectal Ultrasound-Guided Prostatic Needle Biopsy

**DOI:** 10.5812/numonthly.4015

**Published:** 2012-06-20

**Authors:** Mahavir Singh Griwan, Ashok Kumar, Jyotsna Sen, Santosh Kumar Singh

**Affiliations:** 1 Department of Surgery, Pandit B.D.Sharma University of Health Sciences, Rohtak, India; 2 Department of Radiodiagnosis, Pandit B.D. Sharma University of Health Sciences, Rohtak, India; 3 Department of Urology, Pandit B.D. Sharma University of Health Sciences, Rohtak, India

**Keywords:** Pain, Analgesia, Lidocaine

## Abstract

**Background:**

The aim of the present study was to compare two analgesic techniques for transrectal ultrasound (TRUS)-guided biopsy: diclofenac patch versus periprostatic nerve block with 1% lidocaine.

**Objectives:**

To study the efficacy of and compare diclofenac patch and periprostatic nerve block as analgesia in TRUS-guided prostate needle biopsy.

**Patients and Methods:**

In total, 60 patients were prospectively randomized into three groups: those in whom a diclofenac patch was used (n = 20), those in whom periprostatic nerve block was used (n = 20), and a control group (n = 20). Prostate biopsy was performed after administration of analgesia according to group.

**Results:**

The three groups were similar in terms of age, prostate volume, and PSA (prostate-specific antigen) levels. Pain scores were significantly lower in the nerve block group (P = 0.000) at the time of biopsy until 2 h postprocedure, but not at 4 h postprocedure (P = 0.068). No significant difference in pain score was observed in the diclofenac patch group at the time of biopsy (P = 0.106) as compared to the control group, but the diclofenac patch provided adequate pain relief 1 h (P = 0.000), 2 h (0.000), and 4 h (0.002) postprocedure. No significant difference was observed in pain score between the nerve block (P = 0.520) and control groups (0.057) at probe insertion. The pain score at 4 h was significantly lower in the patch group compared to the nerve block and control groups.

**Conclusions:**

Periprostatic nerve block provides superior analgesia for TRUS-guided biopsy. Diclofenac patch is useful as an adjunct.

## 1. Background

Transrectal ultrasound (TRUS)-guided biopsy has become the standard procedure in detection of prostatic carcinoma. Histological diagnosis following TRUS-guided biopsy is vital prior to treatment of prostatic carcinoma. In recent years, a consensus has been reached that sextant sampling is inadequate; sampling with 8 cores or more has been suggested (1). Extended techniques such as TRUS-guided biopsy allow more biopsy samples to be obtained, thereby increasing the prostate cancer detection rate. An increased number of cores translates into increased pain and higher pain scores without analgesia. Kevar et al. labeled this concept “cumulative pain” (2).

Studies show that almost 20% of patients report significant pain associated with biopsy, and that they would refuse rebiopsy without analgesia (1). Men scheduled for TRUS-guided biopsy experience considerable psychological stress. Reasons for this stress include fear of cancer diagnosis, anal route of penetration, sexual anxiety, and anticipated pain associated with the procedure. These minor morbidities are traumatic and worrisome to patients. Anxiety correlates with higher pain scores, especially in younger patients (3) Moderate to severe pain scores were associated with prostate biopsy in the study of Crudwell et al.(4).

TRUS is usually performed in the peripheral zone, which has the highest incidence of prostate carcinoma. Various analgesic modalities during and after TRUS-guided prostate biopsy have been described in the literature, but no one technique has proved most effective. This study compared two modalities of analgesia, periprostatic nerve block and diclofenac patch, to determine the optimal technique for use in TRUS-guided prostate needle biopsy.

## 2. Objectives

To study the efficacy of diclofenac patch and periprostatic nerve block as analgesic in TRUS guided prostate biopsy. To make comparison of diclofenac patch and periprostatic nerve block as analgesia in case of TRUS guided prostate needle biopsy.

## 3. Patients and Methods

This prospective, randomized, controlled trial comparing periprostatic nerve block and diclofenac patch included 60 patients in the Department of Surgery at Pt. B.D.S. University of Health Sciences, Rohtak, India in collaboration with the Department of Urology and Department of Radiodiagnosis in 2010–2011.

Patients with elevated prostate-specific antigen (PSA) levels (>4 ng/mL) and abnormal results on digital rectal examination (DRE), that is, detection of a discrete nodule, focal induration, or diffusely hard prostate, were included in this study. Patients with history of previous biopsy, chronic prostatitis, chronic pelvic pain, inflammatory bowel diseases, anorectal problems, active urinary tract infections, or allergy to local anesthetic were excluded from this study.

### 3.1. Patient Preparation

**Figure 1:**
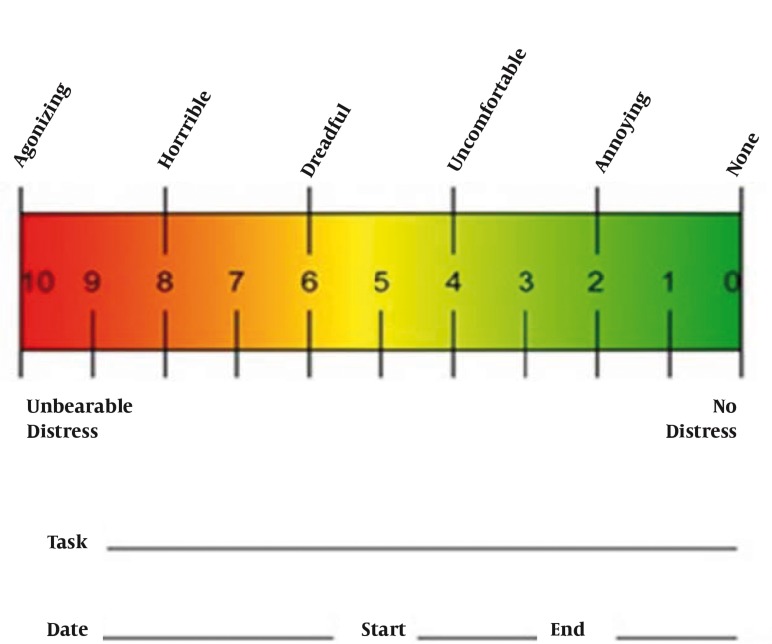
Pain Scale

Patients were prospectively randomized into 1 of 3 groups: those in whom a diclofenac patch was used (n = 20), those in whom periprostatic nerve block was used (n = 20), and a control group (n = 20). In Group A (the diclofenac group), a transdermal patch containing 100 mg of diclofenac was applied over the right forearm 1 h prior to the procedure. In Group B (the nerve block group), a nerve block of 5 mL 1% lidocaine was injected just lateral to the junction between the prostate base and the seminal vesicle immediately prior to biopsy. In Group C (control group), no analgesic was administered. Patients were instructed as to how to describe pain according to the 10-cm visual pain scale depicted in Figure 1. Informed consent was obtained from all patients prior to the procedure.

Phosphatidylcholine enema was administered to all patients 1 h prior to the procedure. Administration of prophylactic antibiotics was initiated 1 day prior to biopsy in the form of ciprofloxacin 500 mg twice a day for 3 days to prevent infection.

Patients were positioned in the left lateral decubitus position for easier insertion of the rectal probe. DRE with lubricating jelly was performed prior to probe insertion to rule out any rectal pathology that would contraindicate the procedure, and to identify any palpable prostatic abnormalities to which special attention should be paid during ultrasound examination.

### 3.2. Nerve Block and Biopsy Procedures

The probe was adjusted to the sagittal plane, and the on-screen biopsy guide was operational before placement. A 22-gauge, 7-inch spinal needle was placed through the biopsy guide channel fitted on the probe under ultrasound guidance into the area where the prostatic innervation enters the gland. The probe was angled laterally until the notch between the prostate and the seminal vesicle was visualized, at which point 1% lidocaine (5 mL) was injected bilaterally. Successful placement of the needle was confirmed when the injectate caused a separation of the seminal vesicles and prostate from the rectal wall (i.e., visualization of the ultrasonic wheal).

The probe was gently advanced into the rectum to the base of the bladder until the seminal vesicles were visualized. Transverse images were then obtained from the prostate base to the prostate apex. With the transducer at the largest cross-sectional area in the transverse plane and mid-sagittal plane, prostate volume was calculated.

An 18-gauge, 20-cm biopsy needle loaded into a spring-action automatic biopsy device was used to procure a minimum of twelve 1.5-cm prostate core biopsy specimens. The needle was introduced through a needle guide fitted over the probe under ultrasound guidance. The area of interest was viewed and biopsies taken under ultrasound guidance. After biopsy, patients were shown the 10-cm visual scale (Figure 1) and asked to describe pain levels at the time of probe insertion and at the time of biopsy according to severity. During the postprocedural rest period, patients were asked to describe pain levels at 1, 2, and 4 h. Responses were scored on the same scale.

### 3.3. Statistical Analysis

Data was analyzed using SPSS version 17.0 (SPSS, Inc., Chicago, IL, USA). Differences in pain scores, age, PSA, and prostate volume in the three groups were analyzed using student’s t test. A two sided P value ≤0.05 was considered significant.

## 4. Results

**Figure 2:**
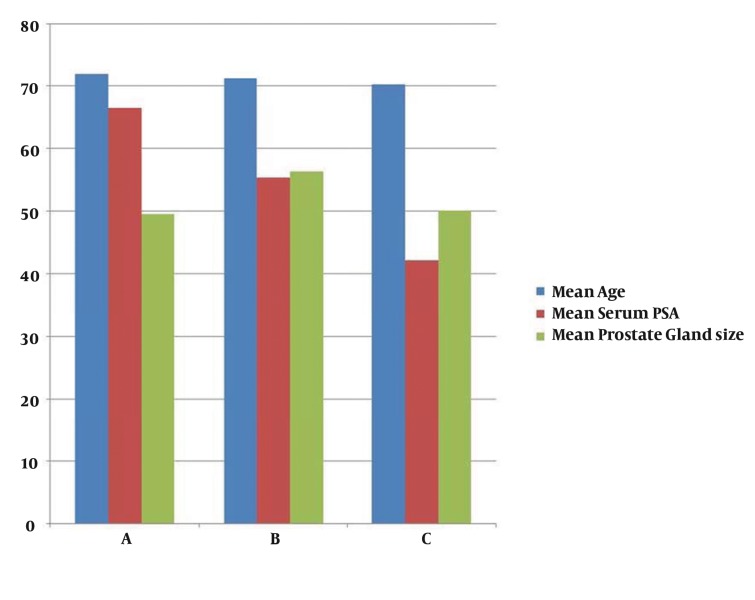
Patient Characteristics

**Figure 3:**
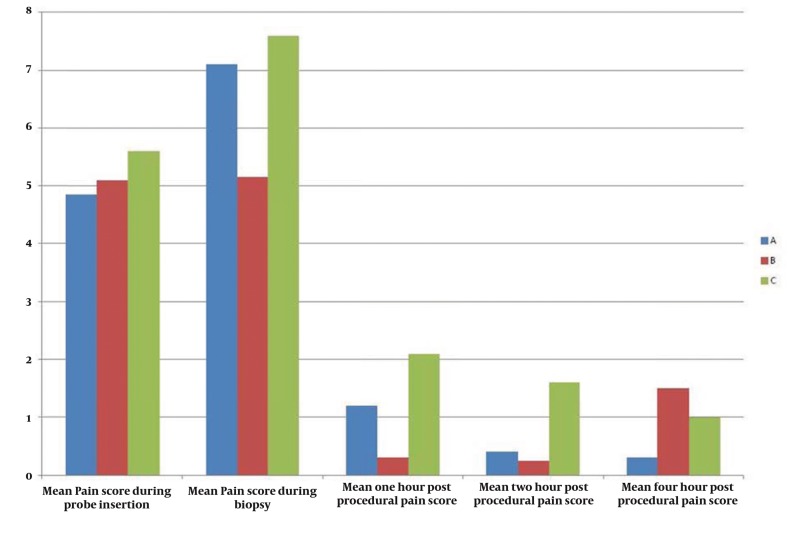
Mean Pain Scores in the Three Groups

No significant differences were found in the three groups in terms of age, prostate volume, and PSA levels (Table 1)(Figure 2). Pain scores were significantly lower in the nerve block group (P = 0.000) at the time of biopsy until 2 h postprocedure, but not at 4 h postprocedure (P = 0.068). No significant difference in pain score was observed in the diclofenac patch group as compared to the control group at the time of biopsy (P = 0.106), but the diclofenac patch provided adequate pain relief 1 h (P = 0.000), 2 h (0.000), and 4 h (0.002) postprocedure. No significant difference was observed in pain score between the nerve block (P = 0.520) and control groups (0.057) at probe insertion (Figure 3). The pain score was significantly higher at 4 h postprocedure in the patch group compared to the nerve block and control groups (Table 2).

**Table 1:** Patient Characteristics by Group (mean ± SD)

	**A**	**B**	**C**
Age, y	71.90 ± 10.427	71.25 ± 10.48	70.30 ± 7.63
Serum PSA^a^, ng/mL	66.55 ± 44.64	55.40 ± 33.40	42.2 ± 35.6
Prostate volume , X	49.53 ± 24.57	56.35 ± 25.64	50.10 ± 21.54

^a^Abbreviation: PSA, prostate specific antigen

**Table 2:** Mean Pain Scores by Group (± SD)

**Time Point**	**A**	***t*value**	**B**	***P* value**	**C**
During probe insertion	4.85± 1.09	0.057	5.10 ± 1.33	0.520	5.60 ± 1.31
During biopsy	7.10 ± 0.85	0.106	5.15 ± 1.50	0.000	7.60 ± 1.05
1 h postprocedure	1.20 ± 0.70	0.000	0.30 ± 0.73	0.000	2.10 ± 0.55
2 h postprocedure	0.40 ± 0.50	0.000	0.25 ± 0.4	0.000	1.60 ± 0.50
4 h postprocedure	0.30 ± 0.47	0.002	1.50 ± 0.89	0.068	1.00 ± 0.80

Complications were similar in all three groups and included bacteremia, rectal bleeding, hematuria, fever, pain during voiding, hematospermia, and urine retention. In groups A, B, and C, 34, 36, and 41 complications were reported, respectively (more than 1 complication per patient was reported in many cases). All complications were minor and managed on an outpatient basis.

## 5. Discussion

Although prostate biopsy has been proven as safe and effective, patients experience significant discomfort during and after the procedure (5). Management of pain associated with TRUS-guided prostate biopsy is important to prevent refusal of rebiopsy in relevant cases.

Pain is a complex perceptual experience that is difficult to quantify. Pain may be a combination of actual somatic and visceral pain, anxiety, and psychological stress; thus, interpretation of pain scores remains subjective. In the report of Desgrandchamps et al., patients were asked to score the severity of postprocedure discomfort using a self-administered verbal rating scale consisting of adjectives describing different levels of pain ranging from “none” to “intolerable pain”(6). This linear 11-point visual analogue scale, which is easily comprehensible and easy to demonstrate, is the most often used scoring method. Others have modified and used this scale in various ways. Scale used as shown in figure.

Pain during prostate biopsy occurs due to insertion of the ultrasound probe into the rectum and needle puncture into the prostate gland. The nerve supply of the prostate is autonomic and originates from the inferior hypogastric plexus. The nerves pass along the plane between the rectum and the prostatic capsule. The pain associated with prostate biopsy is thought to be caused by direct contact of the biopsy needle with these nerves within the stroma and prostatic capsule, which are richly innervated (7). Postprocedural pain may be associated with the production of potent local mediators, such as cytokines, prostaglandins, and leukotrienes, which are associated with edema and the recruitment of immunocompetent cells (8).

Diclofenac acts locally and systemically as an anti-inflammatory agent that decreases the effects of local mediators involved in the pain response. Adiyat et al. advised against use of the diclofenac patch or diclofenac suppository as single agents during prostate biopsy, but suggested they be used as adjunct treatment (9). No increased incidence of complications in the diclofenac treatment group in comparison to control was found in that study. Similar results were found in our study in the diclofenac group.

Ragavan (10) compared three patient groups receiving periprostatic nerve block with 1% lidocaine (10 mL), 100 mg diclofenac suppository, and a combination of both drugs. No significant difference was observed between the three groups in terms of pain at the time of probe insertion, 1 h after biopsy, and on the day after procedure. However, a significant difference in biopsy pain was found in the groups that received the periprostatic nerve block compared to diclofenac alone. Patients in the diclofenac alone group had the lowest analgesic use compared with the combination and lidocaine alone group (14.6% vs. 30.6% and 40% respectively) (10). In our study, significantly lower scores were observed in the nerve block group at the time of biopsy until 2 h postprocedure, while pain scores with the diclofenac patch were significantly higher after the procedure as compared to control.

Wu et al. found no benefit in patient recovery after transrectal biopsy of 5 mL of lidocaine infiltration injected laterally to the seminal vesicles bilaterally compared with placebo (11). However, this result may be explained by the small sample size in that study or insufficient local dose of lidocaine. The current study demonstrated that periprostatic nerve block of 5 mL 1% lidocaine injected just lateral to the junction between the prostate base and the seminal vesicle immediately prior to biopsy provides sufficient analgesia.

Schostak et al. compared four groups of patients in the following analgesic treatment groups: no local anesthesia, anesthetic block of the prostatic plexus, local anesthesia onto the capsule of the apex, and a combination of the two latter methods (12). They found that all types of local anesthesia resulted in lower pain scores. The most effective was apical infiltration, which was technically less difficult to administer than anesthetic block, minimally invasive, and associated with lower morbidity. Taverna et al. utilized a single bolus of 10 mL lidocaine administered at the prostatic midline between Denonvilliers’ fascia and the periprostatic fascia overlying the prostate(13). They found this option to be safe, well-tolerated, and effective with no increase in adverse effects. Similar results were observed in this study in the nerve block group.

Rabets et al. found bupivacaine to be as effective as a lidocaine/bupivacaine combination in providing sufficient immediate anesthesia for transrectal prostate biopsy (14). They injected analgesics into the hyperechoic notch between the prostate and the seminal vesicle, which they termed the “Mount Everest sign”. Further investigation is required to verify this assertion.

Finally, some authors advocate the use of intravenous analgesia with diazepam, cyclizine, and morphine. Others recommend inhalation of 50% nitrous oxide and oxygen or administration of oral analgesics (nonsteroidal anti-inflammatory drugs and opioids). Use of intrarectal enemas with 1% lidocaine has been suggested as equally effective as periprostatic injection of 1% lidocaine for pain control. Other studies promote the use of topical anesthesia (EMLA cream) over placebo and periprostatic nerve blockage with lidocaine (15) Although numerous techniques have been used to reduce or abolish pain in transrectal biopsy, none has been definitively proven as superior to periprostatic nerve blockage and local anesthetic (16).

In the current study, periprostatic nerve block was effective when used as a single anesthetic agent in TRUS-guided prostate biopsy compared to the diclofenac patch at the time of biopsy until 2 h postprocedure. However, the diclofenac patch provided better pain relief 4 h postprocedure. Therefore diclofenac may play a significant role as an adjunct treatment. Perhaps due to their differing mechanisms of action, these techniques used together may provide sufficient analgesia at all time points in and after the procedure.
